# Breast Metastasis from Pulmonary Mucoepidermoid Carcinoma in a Male Patient: A Case Report

**DOI:** 10.3390/curroncol33020094

**Published:** 2026-02-04

**Authors:** Raquel Diaz, Letizia Cuniolo, Rebecca Allievi, Ilaria Baldelli, Federica Murelli, Chiara Cornacchia, Francesca Depaoli, Cecilia Margarino, Chiara Boccardo, Marco Gipponi, Simonetta Franchelli, Marianna Pesce, Giovanni Rossi, Abdallah Saad, Umberto Meliga, Francesca Maria Scura, Santina Petroccelli, Gabriele Puglisi, Emanuela Barisione, Piero Fregatti

**Affiliations:** 1 Department of Surgical Sciences and Integrated Diagnostic (DISC), University of Genoa, 16132 Genoa, Italy; 2 Breast Surgery Unit, IRCCS Ospedale Policlinico San Martino, 16132 Genoa, Italy; 3 Plastic and Reconstructive Surgery Division, IRCCS Ospedale Policlinico San Martino, 16132 Genova, Italy; 4 U.O.C. Medical Oncology 2, IRCCS Ospedale Policlinico San Martino, 16132 Genoa, Italy; 5 Interventional Pulmonology Unit, Division of Respiratory Medicine, IRCCS Ospedale Policlinico San Martino, 16132 Genoa, Italy

**Keywords:** mucoepidermoid carcinoma, lung cancer, salivary gland-type tumor, breast metastasis

## Abstract

Mucoepidermoid carcinoma of the lung is a rare salivary gland-type tumor with heterogeneous clinical behavior and the potential to mimic neoplasms arising in other organs. The purpose of this report is to describe an exceptionally uncommon presentation of pulmonary mucoepidermoid carcinoma manifesting as a breast metastasis in a male patient, a scenario that poses significant diagnostic challenges due to its rarity and its morphological resemblance to primary breast carcinoma. We evaluated the patient through clinical examination, cross-sectional imaging, endobronchial procedures, ultrasound-guided biopsy, immunohistochemistry, and molecular analysis, integrating these data to establish the diagnosis. Imaging revealed a primary lung mass and a second lesion in the left breast infiltrating the pectoralis muscle. Biopsy of the breast mass showed high-grade salivary gland-type mucoepidermoid carcinoma, clinically and radiologically suggestive of pulmonary origin. Because the lesion showed signs of impending ulceration, palliative surgical debulking was performed with good postoperative recovery. The patient subsequently began systemic therapy with gemcitabine. This case underscores the need for careful clinicopathologic correlation when evaluating atypical breast lesions and highlights the diagnostic value of molecular testing in distinguishing primary from metastatic salivary gland-type tumors. Recognizing such rare metastatic patterns is essential for appropriate therapeutic planning.

## Simple Summary

Mucoepidermoid carcinoma of the lung is a very uncommon type of tumor that arises from cells similar to those found in the salivary glands. Although it usually grows slowly, some forms can behave aggressively and spread to distant organs. Metastasis to the breast is exceptionally rare, especially in men, and can be difficult to distinguish from a primary breast cancer because the two conditions may look very similar under the microscope. In this report, we describe the case of an elderly male patient who presented with a breast mass that was ultimately found to originate from a lung tumor. The diagnosis required careful interpretation of imaging studies, detailed histological assessment, and molecular testing. This case highlights the importance of considering unusual metastatic pathways and shows how integrating clinical, pathological, and molecular information can guide appropriate treatment decisions.

## 1. Introduction

Lung cancer remains the leading cause of cancer-related mortality worldwide, reflecting both its high incidence and the typically advanced stage at diagnosis. Within the broad spectrum of pulmonary neoplasms, salivary gland-type tumors represent a distinctly uncommon subgroup that includes mucoepidermoid carcinoma (MEC), adenoid cystic carcinoma, and epithelial–myoepithelial carcinoma [1,2,3,4]. Pulmonary MEC accounts for only 0.1–0.2% of all primary lung neoplasms [1,5] and is believed to arise from the submucosal glands of the tracheobronchial tree. Histologically, these tumors consist of varying proportions of mucous, intermediate, and squamous cells. Molecularly, the presence of CRTC1-MAML2 or, less commonly, CRTC3-MAML2 fusions is a hallmark feature shared with salivary gland tumors [1,2,3,5], providing additional support for a common biological origin across anatomical sites. However, MAML2 rearrangements, while characteristic, are not required for diagnosis and may be unavailable in routine clinical practice.

Although pulmonary MEC is frequently low- to intermediate-grade, high-grade variants may exhibit aggressive behavior, with potential for rapid growth, local invasion, and metastasis [1,4,5,6]. Common metastatic sites include the brain, liver, bone, and adrenal glands, whereas breast involvement is exceptional and accounts for less than 2% of all breast malignancies [2,3,7]. In male patients, this presentation is even rarer, making accurate diagnosis particularly challenging and demanding a meticulous diagnostic approach.

Differentiating between primary breast MEC and metastatic pulmonary MEC can be difficult because of overlapping morphological and immunophenotypic features [7,8]. Even advanced imaging can yield ambiguous findings, especially when lesions display morphologic similarities to primary breast carcinomas. Comprehensive integration of clinical context, imaging, pathology, and molecular diagnostics—particularly demonstration of identical MAML2 rearrangements in both lesions has been reported to support metastatic origin in selected cases, when interpreted within the appropriate clinical context [8,9,10]. However, molecular confirmation may not always be available in routine clinical practice. An accurate diagnosis is essential, as management strategies differ significantly between primary and metastatic salivary gland-type lesions of the breast.

Herein, we report the case of a 79-year-old man with primary pulmonary MEC and synchronous metastatic involvement of the left breast. This case highlights the diagnostic complexities associated with such a rare presentation and emphasizes the critical role of molecular characterization in distinguishing primary from metastatic disease. Additionally, it underscores the importance of recognizing atypical metastatic routes, which can have important implications for prognosis and clinical management.

## 2. Case Presentation

A 79-year-old man presented with new-onset hemoptysis and a palpable mass in the upper quadrants of the left breast. His past medical history was unremarkable for thoracic malignancies or breast disease. The patient reported progressive enlargement of the breast mass over several weeks, accompanied by localized discomfort and erythema, raising initial concern for an inflammatory or malignant process.

A contrast-enhanced chest CT scan revealed a solid intraparenchymal lesion measuring 42 × 35 mm in the upper lobe of the left lung. A second lesion, measuring 62 mm, was visualized in the upper quadrants of the left breast, with radiological evidence of infiltration into the pectoralis major muscle. Targeted breast ultrasound confirmed the solid nature and deep extension of the mass, further supporting suspicion for malignancy.

An EBUS-guided cryobiopsy of the pulmonary lesion revealed no malignant cells. EBUS-guided TBNA of a lymph node at station 11 L also showed no evidence of malignancy. Despite the absence of diagnostic tissue from the pulmonary biopsies, the radiologic findings remained concerning for a high-grade primary lung tumor.

Ultrasound-guided core biopsy of the breast lesion showed a salivary gland-type MEC with glandular and squamous differentiation. Immunohistochemistry demonstrated positivity for CK7 and p63, focal expression of CK5/6, and weak expression of E-cadherin. CK20, GATA-3, ER, PR, HER2, CDX2, TTF-1, Napsin-A, GCDFP-15, p40, and SOX10 were negative. Molecular testing revealed a PIK3CA mutation and low PD-L1 expression.

Staging with brain and abdominal CT scans revealed no distant metastases. PET/CT demonstrated intense FDG uptake in both the lung lesion (SUVmax 23.1) and breast mass (SUVmax 25), strongly suggestive of metabolically active malignancy in both locations.

**Figure 1:**
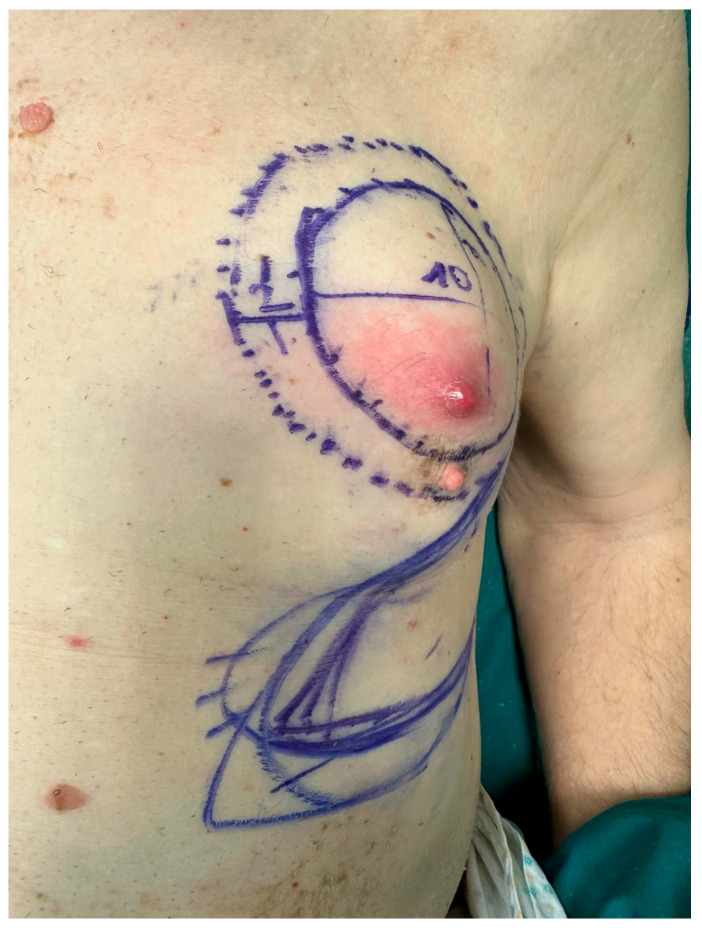
Preoperative photograph showing the left breast mass prior to surgical intervention.

**Figure 2:**
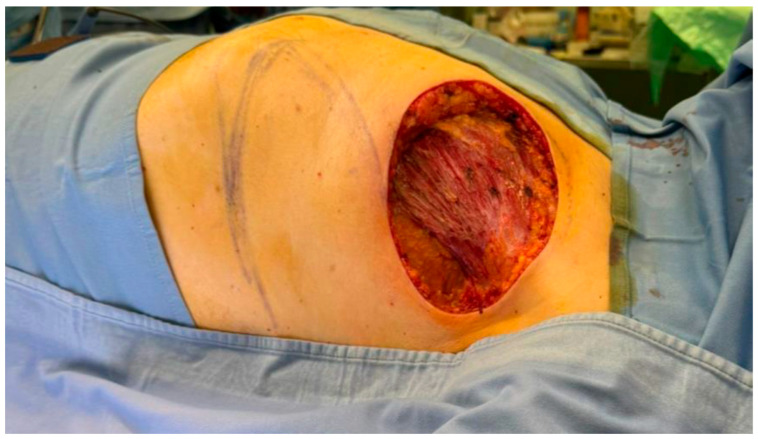
Intraoperative planning of the TA flap following demolition surgery.

Given signs of imminent ulceration of the breast lesion, palliative surgical debulking was indicated (Figure 1). The patient underwent resection of the nipple–areolar complex and partial excision of the pectoralis major muscle. Reconstruction was performed using a thoracoepigastric fasciocutaneous flap, selected to achieve rapid and reliable wound closure and to allow early postoperative recovery and timely initiation of adjuvant therapy [11]. Following tumor resection, the flap was planned according to the size of the surgical defect and designed with a lateral pedicle (Figure 2).

**Figure 3:**
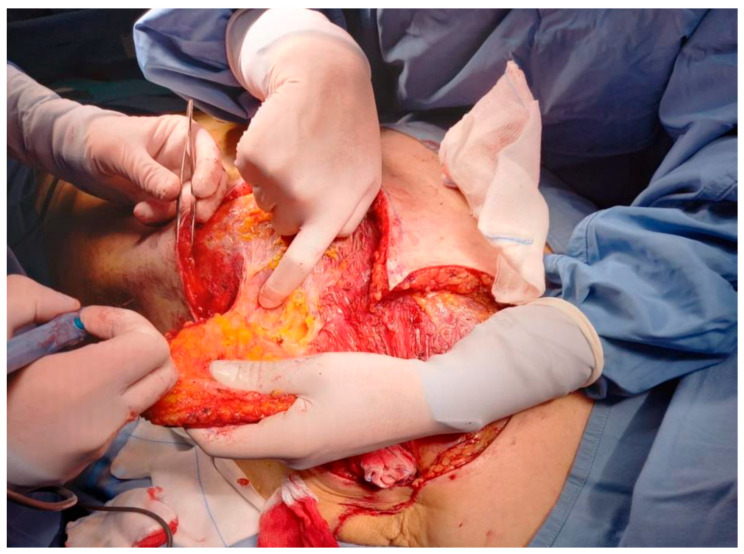
Identification of perforating arteries.

**Figure 4:**
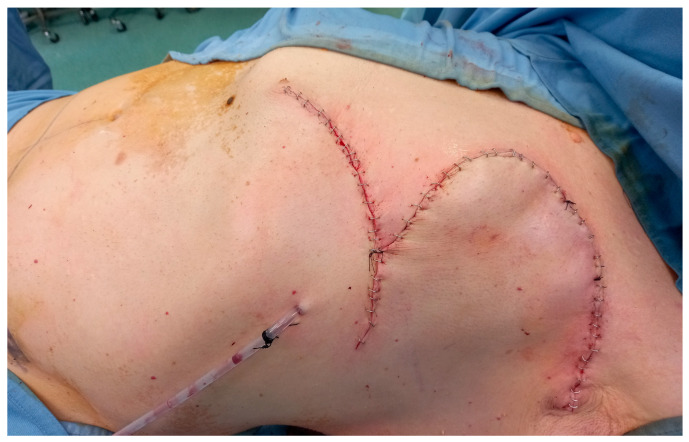
Immediate postoperative appearance.

**Figure 5:**
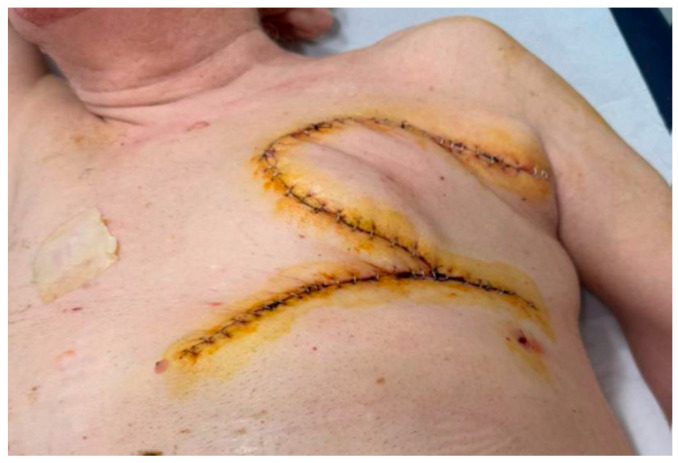
Five days postoperative follow-up.

Elevation of the flap was performed in the subfascial plane, with careful identification and preservation of the perforating vessels to ensure adequate vascularization (Figure 3). The flap was then transposed to the defect and inset with minimal tension, achieving satisfactory coverage (Figure 4). The donor site was closed directly, and the postoperative course was uneventful, with good wound healing and no reconstructive complications observed at early follow-up (Figure 5). This technique provides well-vascularized tissue coverage with shorter healing times and lower morbidity compared with skin grafts, particularly in patients requiring early systemic therapy [12,13].

The postoperative course was uneventful. The patient was discharged on postoperative day 2, and the drain was removed on postoperative day 6. Final histology confirmed high-grade salivary gland-type MEC. Based on the overall clinical and radiological context, the lung was considered the most likely primary site. Margins were negative, and no additional actionable mutations were identified.

During outpatient wound checks, the patient developed a small wound dehiscence, which was successfully managed with conservative treatment.

The patient is currently in good general condition, with complete wound healing. He is receiving gemcitabine on days 1 and 8 of a 21-day cycle and continues to be followed clinically and radiologically at regular intervals. Single-agent gemcitabine was selected in consideration of the patient’s advanced age, comorbidities, and palliative treatment intent, with the aim of balancing disease control and treatment tolerability.

All the important examinations, diagnoses, and therapies are summarized in a timeline (Table 1).

## 3. Discussion

Breast metastases originating from extramammary malignancies are rare and represent less than 2% of all breast tumors. Among pulmonary neoplasms, adenocarcinoma and small-cell carcinoma are the most frequent histotypes associated with breast metastases, while mucoepidermoid carcinoma is extraordinarily uncommon in this context. The rarity is even more pronounced in male patients, making this case clinically exceptional. Pulmonary MEC, although accounting for only 0.1–0.2% of lung neoplasms, is characterized by a mixture of mucous, intermediate, and squamous cells and by recurrent CRTC1-MAML2 fusions [1,2,3,4,5]. High-grade MEC may pursue an aggressive trajectory with potential for local invasion and distant spread, as observed in the present case.

Distinguishing metastatic pulmonary MEC from primary breast MEC can be challenging because of overlapping cytoarchitectural and immunophenotypic characteristics [2,3,4,7,8]. Histology alone is often insufficient, as both primary and metastatic variants may show similar patterns of squamous and mucinous differentiation. Immunohistochemistry can help narrow the diagnostic possibilities but does not provide a definitive distinction, given the shared expression of markers such as p63, CK7, and CK5/6. Therefore, histology and immunohistochemistry alone cannot reliably distinguish primary breast MEC from metastatic pulmonary MEC. For this reason, the interpretation of such cases requires careful integration of pathological findings with clinical presentation and imaging features.

In this context, molecular testing represents a valuable complementary diagnostic tool. The presence of MAML2 rearrangements has been widely described in MEC across different anatomical sites [9,10]. While demonstration of molecular identity between two lesions may support a metastatic relationship, MAML2 rearrangements are not site-specific and may occur in both pulmonary and breast MEC. Accordingly, molecular findings should be interpreted in conjunction with clinical, radiological, and pathological features, and no single modality is sufficient to establish tumor origin with certainty.

The management of metastatic MEC remains largely empirical due to its rarity and the absence of standardized therapeutic algorithms. Systemic therapy choices often rely on extrapolation from salivary gland MEC or general principles applied to high-grade thoracic tumors. The identification of a PIK3CA mutation in our patient is noteworthy, as alterations in the PI3K/AKT/mTOR pathway are increasingly recognized in salivary gland-type tumors and may theoretically offer opportunities for targeted therapy. However, evidence supporting PI3K inhibitors in pulmonary MEC is limited to anecdotal reports, and no approved targeted agents currently exist for this disease. Similarly, the relevance of immunotherapy remains uncertain. PD-L1 expression in MEC is generally low, and these tumors often display a “cold” immune microenvironment, which correlates with poorer responses to immune checkpoint inhibitors compared to other non-small-cell lung cancer subtypes. In this patient, low PD-L1 levels reinforced the expectation of limited benefit from immunotherapy.

The rarity of breast metastasis from pulmonary MEC also has important implications for clinical evaluation. Misinterpretation as a primary breast carcinoma is a recognized risk, particularly when evaluating lesions that share morphological similarities with high-grade triple-negative breast cancers. This risk is further amplified in male patients, in whom primary breast malignancies are themselves infrequent. Such scenarios underscore the importance of integrating clinical history, imaging, pathology, and molecular diagnostics to avoid misclassification, which would significantly alter therapeutic planning. Additionally, given the potential for misleading radiologic and pathologic findings, multidisciplinary input is crucial to ensure accurate diagnosis and optimal treatment selection. A summary of published studies on MEC involving the breast or the lung is shown in Table 2.

In our case, the decision to proceed with palliative surgical debulking was driven by local symptoms and the imminent risk of ulceration. Surgical management allowed for rapid symptom relief, prevented complications, and facilitated subsequent systemic therapy. Gemcitabine was selected as palliative treatment based on available clinical experience in high-grade salivary gland-type tumors, although robust evidence remains lacking [1,6]. As with many rare malignancies, management must be individualized, considering molecular findings, tumor burden, patient comorbidities, and symptom control.

Overall, this case highlights the need for multidisciplinary decision-making and individualized treatment strategies in the management of rare metastatic patterns associated with pulmonary MEC. It also contributes to the limited but growing body of literature on unusual metastatic behaviors of salivary gland-type lung tumors, reinforcing the essential role of molecular diagnostics in contemporary oncologic practice.

## 4. Conclusions

Breast metastasis from pulmonary MEC is exceedingly rare, particularly in male patients. Accurate diagnosis requires careful integration of clinical, radiologic, histopathologic, and molecular data. Although molecular alterations such as MAML2 rearrangements may support a metastatic relationship, they are not site-specific and must be interpreted in conjunction with clinical and radiological findings. In this case, the diagnosis relied on integrated clinicopathologic evaluation rather than definitive molecular proof.

Given the absence of standardized treatment, management should be individualized. Palliative surgery may be appropriate for symptomatic lesions, and systemic therapy should be tailored to tumor biology. This case underscores the importance of maintaining suspicion for metastatic disease when evaluating atypical breast lesions—even in male patients—and highlights the crucial role of molecular diagnostics in challenging oncologic scenarios.

## Figures and Tables

**Table 1:** Clinical timeline.

Phase	Summary
Presentation	Hemoptysis and palpable left breast mass
Initial Imaging	CT scan: 42 × 35 mm left upper lobe lesion; 62 mm breast mass infiltrating pectoralis
EBUS Procedures	Cryobiopsy and TBNA negative for malignancy
Breast Biopsy	High-grade salivary gland-type MEC, IHC supporting pulmonary origin
Staging	PET/CT scan: intense uptake both lung and breast lesions; no other metastases
Preoperative Phase	Ulceration risk → surgical indication
Surgery	Palliative debulking + NAC excision + partial pectoralis major resection + flap reconstruction
Postoperative	Uneventful, drain removed day 6
Final Histology	High-grade MEC consistent with pulmonary origin
Current Treatment	Gemcitabine (day 1 & 8 every 21 days)
Current Status	Good general conditions, healed wound

**Table 2:** Summary of published studies on MEC involving the breast.

Authors and References	Study Design	Primary Tumor Site	Main Findings
He et al., 2023 [14]	Case report + review	Breast	Three cases of primary breast MEC; no metastasis from lung
Akbulut et al., 2025 [15]	Systematic review	Breast	Review of primary breast MEC; no metastasis from lung
Bak et al., 2022 [16]	Case report + review	Breast	Imaging of primary breast MEC; no metastasis from lung
Li et al., 2022 [17]	Retrospective study	Lung	Prognostic analysis of pulmonary MEC; no breast metastases
Hu et al., 2022 [1]	Review	Lung	Review on pulmonary MEC; no breast metastases
Cheng et al., 2017 [18]	Case report + review	Breast	Four cases of primary breast MEC; no metastasis from lung
Shen et al., 2014 [19]	Retrospective study	Lung	Clinical analysis of pulmonary MEC; no breast metastases
Wang et al., 2024 [20]	Case report + review	Breast	High-grade primary breast MEC; no metastasis from lung
Hsieh et al., 2017 [21]	Retrospective study	Lung	Surgical outcomes of pulmonary MEC; no breast metastases

## Data Availability

The data presented in this study are available in this article.
